# Machine Learning Models for Mortality Prediction in Intensive Care Unit Patients With Ischemic Stroke Associated With Intracranial Artery Stenosis: Retrospective Cohort Study

**DOI:** 10.2196/82042

**Published:** 2026-02-24

**Authors:** Kun Zhang, Ruomeng Chen, Jingyi Yang, Yan Yan, Lijuan Liu, Chaoyue Meng, Peifang Li, Guoying Xing, Xiaoyun Liu

**Affiliations:** 1 The First Hospital of Hebei Medical University, No. 89, Donggang Rd, Yuhua District, Shijiazhuang, Hebei, China, 86 13191887318; 2 The Second Hospital of Hebei Medical University, Shijiazhuang, Hebei, China; 3 Handan Central Hospital, Handan, Hebei, China; 4 Zhaoxian Traditional Chinese Medicine Hospital, Shijiazhuang, Hebei, China

**Keywords:** ischemic stroke, intracranial arterial stenosis, critical care, machine learning, mortality prediction, explainable artificial intelligence, intensive care unit, ICU outcomes

## Abstract

**Background:**

Mortality prediction in intensive care unit (ICU) patients with ischemic stroke complicated by intracranial artery stenosis or occlusion remains difficult. Conventional scoring systems often lack discriminatory power and fail to provide individualized risk estimates. Machine learning approaches have been increasingly explored to integrate diverse clinical features for prognostic modeling.

**Objective:**

This study aims to develop and evaluate machine learning models for individualized mortality prediction in ICU patients with ischemic stroke associated with intracranial artery stenosis or occlusion.

**Methods:**

Using the Medical Information Mart for Intensive Care IV (MIMIC-IV) database, we conducted a retrospective cohort study including 5280 adult ICU patients identified through *International Classification of Diseases, Ninth and Tenth Revision* (*ICD-9/10*) codes. Mortality status was determined based on the presence of a recorded date of death (dod) in the MIMIC-IV database. Patients with a documented dod were classified as deceased, whereas those without a recorded dod were classified as nondeceased. The primary outcome was all-cause mortality as recorded in the MIMIC-IV database, defined by the presence of a documented dod. Patients were randomly split into training (n=3696, 70%) and testing (n=1584, 30%) cohorts. Missing value imputation, correlation reduction, and multistep supervised feature selection (gradient boosting, BorutaShap, recursive feature elimination with cross-validation, LassoCV, and chi-square analysis) were performed exclusively within the training set and subsequently applied to the test set, resulting in 35 retained predictive features. Eight machine learning models—including light gradient boosting machine (LightGBM), Bagging (bootstrap aggregating), random forest, logistic regression, support vector machine, gradient boosting, adaptive boosting, and k-nearest neighbors—were trained with hyperparameter optimization using RandomizedSearchCV. Model performance was evaluated using area under the curve, accuracy, recall, precision, *F*_1_-score, and calibration curves. Shapley additive explanations were used for global and individual-level interpretability.

**Results:**

LightGBM, Bagging, and logistic regression demonstrated comparable discrimination, achieving an area under the curve of approximately 0.82‐0.83 and accuracy above 73% on the independent test set. LightGBM demonstrated balanced performance (recall 0.70; precision 0.72) and good calibration. Shapley additive explanations analysis identified acute physiology score III, suspected infection, Charlson comorbidity index, age, weight on admission, and red cell distribution width as the most influential predictors. Overall, higher physiological severity, greater comorbidity burden, and older age were consistently associated with increased observed mortality risk.

**Conclusions:**

Machine learning models—including LightGBM and Bagging—provide interpretable predictions of all-cause mortality in ICU patients with ischemic stroke and intracranial arterial disease. These models highlight key prognostic features and may support mortality risk stratification. External validation and evaluation of workflow integration are warranted before clinical implementation.

## Introduction

Cerebrovascular diseases, particularly those associated with intracranial artery stenosis or occlusion, are major contributors to morbidity and mortality among critically ill patients [1]. In the intensive care unit (ICU), these conditions often lead to acute neurological deterioration, secondary complications, and poor clinical outcomes, posing significant management challenges. Despite the widespread use of traditional clinical scoring systems, accurate and individualized mortality prediction remains difficult.

Recent advances in machine learning (ML) offer promising approaches to address this challenge [2]. ML algorithms can process large-scale, high-dimensional clinical data to detect complex, nonlinear relationships that conventional methods may overlook [3]. Such tools have shown potential in critical care settings, particularly for mortality prediction, risk stratification, and outcome modeling in severe illnesses including stroke and sepsis.

The Medical Information Mart for Intensive Care IV (MIMIC-IV) database [4] is a publicly accessible ICU dataset containing detailed records of over 350,000 ICU admissions, including demographics, diagnoses, physiological measurements, laboratory tests, and treatments. This rich resource facilitates the development of robust, data-driven predictive models [5].

In this study, we leveraged the MIMIC-IV database to develop and evaluate ML models for predicting in-hospital and post-discharge mortality in ICU patients diagnosed with ischemic stroke associated with intracranial artery stenosis or occlusion. We used advanced feature selection techniques—including gradient boosting classifier, BorutaShap, recursive feature elimination with cross-validation (RFECV), and LassoCV—to optimize the input variables [6]. Additionally, Shapley additive explanations (SHAPs) were integrated to enhance model interpretability and transparency, providing insight into the contribution of each feature to individual predictions [7].

Although several ML-based models have been developed for predicting outcomes in patients with ischemic stroke [8-10], most existing studies have focused on general stroke cohorts or emergency department populations and have rarely examined critically ill patients with intracranial artery stenosis or occlusion. Moreover, prior models typically target in-hospital mortality alone, rely on limited variable sets, or lack transparent feature-interpretation methods. In contrast, our study incorporates a large and granular ICU cohort from the MIMIC-IV database, applies a multistep feature selection pipeline, evaluates multiple ML algorithms under a unified framework, and uses SHAP to provide clinically interpretable insights. By including both in-hospital and postdischarge deaths recorded in the database, our model provides a pragmatic assessment of observed all-cause mortality risk in ICU patients, reflecting real-world outcome documentation in large critical care registries.

Our aim was to establish an interpretable and high-performing prognostic model to support timely and informed clinical decision-making for ICU patients with ischemic stroke. The following sections describe our methodology, present the results, and discuss the clinical implications and future research directions.

## Methods

### Study Design and Data Source

This retrospective cohort study was conducted using the publicly available MIMIC-IV database, which contains detailed clinical data from over 350,000 ICU admissions at the Beth Israel Deaconess Medical Center between 2008 and 2019. The database includes a comprehensive range of variables, such as demographic characteristics, vital signs, laboratory results, diagnoses, medications, and procedures.

Mortality outcomes were ascertained using the publicly available MIMIC-IV database (version 3.1; released on October 11, 2024). Mortality status was determined based on the presence of a recorded date of death (dod) in the database. Patients with a documented dod were classified as deceased, whereas patients without a recorded dod were classified as alive. This definition captures all-cause in-hospital mortality as well as postdischarge mortality when documented in MIMIC-IV. The primary outcome was all-cause mortality, defined by the presence of a recorded dod in the MIMIC-IV database. Patients with a documented dod were classified as deceased, whereas those without a recorded dod were classified as nondeceased. No additional time-window restriction was applied.

This study focused on adult ICU patients diagnosed with ischemic stroke associated with intracranial artery stenosis or occlusion. Patients were identified using *International Classification of Diseases, Ninth and Tenth Revision* (*ICD-9/10*) codes corresponding to cerebrovascular pathology, specifically: I66., I630-I637, 433., 434.*, 437.5, and 437.6. Because cohort selection was not restricted to the principal diagnosis, the study population included critically ill patients in whom ischemic stroke may have been either the primary diagnosis or a significant comorbid condition.

A total of 8437 patient records were initially identified with relevant cerebrovascular diagnoses. To ensure data integrity and minimize redundancy, only the first ICU admission for each patient was considered. After excluding individuals with multiple admissions or incomplete records, the final study cohort comprised 5280 patients aged 18 years or older.

**Figure 1.**
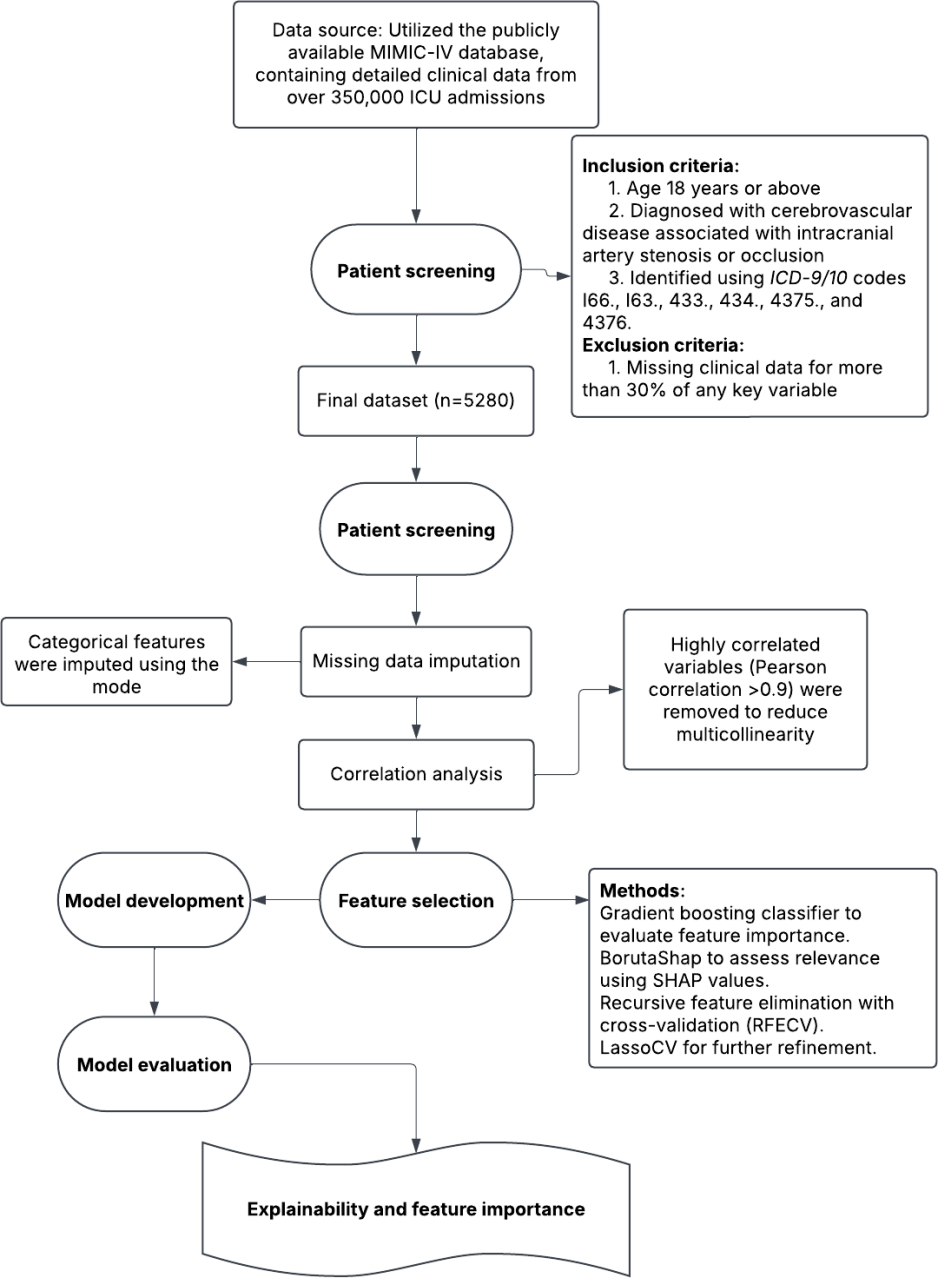
Flowchart of the study design and methodology. *ICD-9/10*: *International Classification of Diseases, Ninth and Tenth Revision*; ICU: intensive care unit; MIMIC-IV: Medical Information Mart for Intensive Care IV; SHAP: Shapley additive explanations.

The primary outcome was all-cause mortality, defined by the presence of a recorded dod in the MIMIC-IV database. The patient selection process is illustrated in Figure 1.

### Inclusion and Exclusion Criteria

Patients were included if they met the following criteria: (1) aged 18 years or older; (2) diagnosed with ischemic stroke associated with intracranial artery stenosis or occlusion, identified using *ICD-9/10* codes from MIMIC-IV, including I66., I630-I637, 433., 434., 437.5, and 437.6; and (3) had documented mortality status based on the presence or absence of a recorded dod in the MIMIC-IV database. From all 364,627 patients in MIMIC-IV, a total of 8437 hospitalization records fulfilled the cerebrovascular diagnostic criteria. For patients with multiple admissions, only the first ICU admission was retained to avoid duplication, resulting in a final cohort of 5280 adult ICU patients.

### Outcome Definition

The primary outcome was observed all-cause mortality, defined by the presence of a recorded dod in the MIMIC-IV database. This outcome reflects mortality events documented during hospitalization and after discharge when available. The absence of a recorded dod was interpreted as survival within the observation window. This definition was applied consistently across the entire cohort and all modeling stages.

### Variables and Features

A comprehensive set of variables was included, comprising:

- Demographic variables: age, sex, height, and weight.
- Clinical parameters: vital signs (eg, heart rate, blood pressure, respiratory rate), laboratory findings (eg, hemoglobin, white blood cell count, glucose, calcium, creatinine).
- Scoring systems: sequential organ failure assessment, acute physiology and chronic health evaluation III (APS-III), and Glasgow Coma Scale.
- Clinical history: Clinical history variables included the Charlson comorbidity index, presence of malignancy, suspected infection (defined algorithmically based on antibiotic administration and body fluid culture collection), use of mechanical ventilation, and pharmacological treatments (eg, anticoagulants and antihypertensive agents).

To ensure adequate data quality, any variable with more than 30% missing values was excluded from further analysis. This criterion was applied to variables only, and no patients were excluded based on this step. Following preprocessing, 151 features were retained for further analysis. To ensure early risk stratification and avoid look-ahead bias, all predictive features were extracted exclusively from the early phase of ICU admission. Demographic characteristics were obtained at baseline, and all laboratory variables were defined as the first available measurement after ICU admission. Physiological parameters, clinical scores, and treatment-related indicators (including mechanical ventilation status and suspected infection) were derived from data recorded within the first 24 hours following ICU admission. No information collected beyond this predefined early time window was used for feature construction, model training, or prediction.

### Data Preprocessing and Feature Selection

To minimize the risk of data leakage, all preprocessing and feature selection procedures were conducted strictly within the training data only.

The dataset was first randomly split into a training set (n=3696, 70%) and an independent testing set (n=1584, 30%). Missing data imputation was performed by fitting the imputation strategy exclusively on the training set. Continuous variables were imputed using the median values derived from the training data to reduce the influence of outliers, while categorical variables were imputed using the mode. The fitted imputation parameters were then applied unchanged to the testing set.

To reduce multicollinearity, Pearson correlation analysis was conducted within the training set. Features with a correlation coefficient greater than 0.9 were excluded, including variables such as “dbp,” “hematocrit,” “rbc,” and “mechvent_score.” The same feature exclusion rules were subsequently applied to the testing set.

Feature selection was performed using a multistep strategy applied exclusively to the training data. This process included (1) gradient boosting classifier–based feature importance ranking; (2) BorutaShap, which integrates SHAP values with the Boruta algorithm for robust supervised feature selection; (3) RFECV; (4) LassoCV for regularization-based feature shrinkage; and (5) chi-square tests to assess statistical associations (*P*<.05).

To enhance robustness and reduce method-specific bias, a consensus feature selection strategy was adopted. Only features consistently selected across multiple methods were retained. This approach resulted in the identification of 35 predictive features with the highest and most stable importance, including “inr_max,” “calcium,” “spo2,” “oasis_x,” “suspected_infection,” and “anchor_age.” The final selected feature set was fixed after training and applied unchanged to the independent testing set.

### Validation Strategy

In addition to the conventional random split, we performed a temporal validation to assess model generalizability across different calendar periods and to minimize potential information leakage due to long study duration. Specifically, patients were grouped according to the MIMIC-IV anchor_year_group variable. Admissions from earlier calendar periods were used for model training, while more recent admissions were reserved for testing. This temporal split mimics a real-world deployment scenario, in which models trained on historical data are applied to future patients. All preprocessing steps, feature selection procedures, and hyperparameter tuning were performed exclusively within the temporally defined training set, and the finalized models were evaluated on the temporally independent test set using the same performance metrics as in the random split analysis.

### Model Development and Hyperparameter Optimization

The training set was used exclusively for model development and hyperparameter tuning, while the testing set was reserved for final performance evaluation. Prior to model training, feature standardization was performed using *z*-score normalization, with scaling parameters estimated from the training data and subsequently applied to the testing data.

Multiple ML classifiers were trained, including light gradient boosting machine (LightGBM), Bagging (bootstrap aggregating), k-nearest neighbors, logistic regression, support vector machine (SVM), random forest, adaptive boosting (AdaBoost), and gradient boosting.

Hyperparameter optimization for each model was conducted using RandomizedSearchCV within the training set, using cross-validation to identify optimal parameter combinations. This training-only optimization strategy ensured that no information from the testing data influenced model selection or tuning. All cross-validation procedures were conducted exclusively within the training set.

Model performance was evaluated using several metrics, including:

- *F*_1_-score: The harmonic mean of precision and recall.
- Precision: The proportion of predicted positives that were positive.
- Recall: The proportion of actual positives that were correctly identified.
- Area under the receiver operating characteristic curve (ROC AUC): A performance measurement for classification problems at various threshold settings.
- Accuracy: The proportion of correctly classified instances.

The performance of each model was visualized through ROC curves to assess classification ability. Confusion matrices were also generated to assess the number of true positives, false positives, true negatives, and false negatives for each model. Calibration performance was assessed using both calibration curves and quantitative metrics, including the Brier score, calibration slope, and calibration intercept, to provide a comprehensive evaluation of the reliability of predicted probabilities.

### Decision Curve Analysis

To evaluate the clinical utility and net benefit of the predictive models, we performed decision curve analysis (DCA). DCA quantifies the net benefit of using a model to guide clinical decisions across a range of threshold probabilities, which reflects the trade-off that clinicians and patients are willing to make between the benefits of a true positive and the harms of a false positive. The net benefit was calculated for our top-performing models (LightGBM, Bagging, extreme gradient boosting (XGBoost), random forest, and AdaBoost) and compared against 2 default strategies: “treat all” (assuming all patients are classified as high risk) and “treat none” (assuming no patients will die). A model with a higher net benefit across a range of thresholds is considered to have greater clinical utility.

### Model Interpretability

To interpret model predictions, we applied SHAP, which quantifies the contribution of each feature to individual predictions. SHAP summary and scatter plots were generated for the best-performing model, LightGBM, offering visual insights into key mortality predictors.

### Statistical Analysis

Chi-square tests were used to determine associations between categorical features and mortality, with a significance level of *P*<.05. The Kolmogorov-Smirnov test was applied to compare the distribution of continuous features between survival and mortality groups.

### Ethical Considerations

This study used the data from the publicly available and fully deidentified MIMIC-IV database. Because the dataset contains no identifiable private information and all patient data are anonymized, this retrospective analysis is exempt from institutional review board approval. The use of MIMIC-IV data complies with all relevant ethical guidelines and the data use agreement required for access to the database. No direct patient contact or intervention occurred, and therefore informed consent was not required.

## Results

### Patient Characteristics

A total of 5280 ICU patients diagnosed with cerebrovascular disease and intracranial artery stenosis or occlusion were included. The cohort had a mean age of 69.1 years (SD 14.6), and 52.4% (n=2765) were male. Table 1 summarizes key demographic, clinical, and laboratory characteristics.

**Table 1.** Demographic, clinical, and laboratory characteristics stratified by mortality status (recorded death date vs no recorded death date)^a^.

	Total (N=5280)	No recorded date of death (n=2952)	Recorded date of death (n=2328)	*P* value
Demographic characteristics				
Age (y), mean (SD)	69.09 (14.61)	66.09 (14.92)	72.89 (13.26)	<.001
Male, n (%)	2765 (52.4)	1178 (50.6)	1587 (53.8)	.02
Female, n (%)	2515 (47.6)	1150 (49.4)	1365 (46.2)	.02
Race or ethnicity, n (%)				
White	3495 (66.2)	1973 (66.8)	1522 (65.4)	—^b^
Black	533 (10.1)	264 (8.9)	269 (11.6)	—
Hispanic or Latino	156 (3)	95 (3.2)	61 (2.6)	—
Asian	144 (2.7)	82 (2.8)	62 (2.7)	—
Other or unknown	952 (18)	538 (18.2)	414 (17.8)	—
Comorbidities				
Hypertension, n (%)	1655 (31.3)	959 (32.5)	696 (29.9)	.05
Diabetes mellitus, n (%)	1799 (34)	874 (29.6)	925 (39.7)	<.001
Dyslipidemia, n (%)	1964 (37.2)	797 (34.2)	1167 (39.5)	<.001
Suspected infection, n (%)	3874 (73.4)	1876 (63.6)	1998 (85.8)	<.001
Dementia, n (%)	251 (4.8)	79 (2.7)	172 (7.4)	<.001
Malignant cancer, n (%)	438 (8.3)	118 (4)	365 (15.7)	<.001
Charlson comorbidity index, median (IQR)	6 (4-8)	5 (3-7)	7 (5-9)	<.001
Laboratory parameters				
LDL-C^c^ (mg/dL), mean (SD)	85.8 (33.2)	88.6 (34.2)	82.3 (31.5)	<.001
Triglycerides (mmol/L), mean (SD)	131.3 (128.8)	130.0 (117.7)	133.0 (141.5)	.12
Blood glucose (mg/dL), mean (SD)	134.1 (60.2)	127.6 (53.0)	142.3 (67.5)	<.001
RDW^d^	14.8 (2.1)	14.4 (1.9)	15.4 (2.3)	<.001
BUN^e^	24.8 (18.5)	20.5 (13.6)	30.3 (22.1)	<.001
Clinical scores				
SOFA^f^ score, mean (SD)	1.5 (1.9)	1.3 (1.8)	1.8 (2.1)	<.001
APS-III^g^ score, mean (SD)	42.7 (19.9)	36.6 (16.1)	50.4 (21.4)	<.001
SAPS-II^h^, mean (SD)	36.4 (13.2)	32.1 (11.3)	41.8 (13.4)	<.001
GCS^i^, mean (SD)	13.8 (2.2)	14.2 (2.0)	13.7 (2.5)	<.001
In-hospital mortality				
Mortality, n (%)	669 (12.7)	0 (0)	669 (28.7)	<.001

a Race or ethnicity was reported as recorded in the electronic health record and aggregated for descriptive purposes only.

b Not available.

c LDL-C: low-density lipoprotein cholesterol.

d RDW: red cell distribution width.

e BUN: blood urea nitrogen.

f SOFA: sequential organ failure assessment.

g APS-III: acute physiology and chronic health evaluation III.

h SAPS II: Simplified Acute Physiology Score II.

i GCS: Glasgow Coma Scale.

The most common comorbidities were hypertension (n=1655, 31.3%), diabetes mellitus (n=1799, 34%), and dyslipidemia (n=1964, 37.2%). Based on the MIMIC-IV recorded dod, 2328 (44.1%) patients were classified as deceased, while 2952 (55.9%) patients had no recorded dod and were classified as nondeceased. The in-hospital mortality rate was 12.7% (n=669), representing a subset of deaths captured by the recorded dod in the MIMIC-IV database.

Compared with patients classified as nondeceased, those classified as deceased were significantly older (mean age: 72.9, SD 13.3 y vs mean age: 66.1, SD 14.9 y; *P*<.001) and had higher Charlson comorbidity index scores (median 7, IQR 5-9 vs median 5, IQR 3-7; *P*<.001). They also had higher incidences of suspected infection (1998/2328, 85.8% vs 1876/2952, 63.6%; *P*<.001) and dementia (172/2328, 7.4% vs 79/2952, 2.7%; *P*<.001). Group differences were observed for laboratory variables such as low-density lipoprotein cholesterol and red cell distribution width (both *P*<.001), whereas triglycerides did not differ significantly (*P*=.12).

### Feature Selection

Following imputation and correlation filtering, 107 features were included. After applying the feature selection pipeline (gradient boosting, BorutaShap, RFECV, LassoCV, and Chi-square testing), 35 features were consistently identified as significant. These features were retained for model development. The full list of selected features is available in Multimedia Appendix 1.

### Model Performance

**Figure 2.**
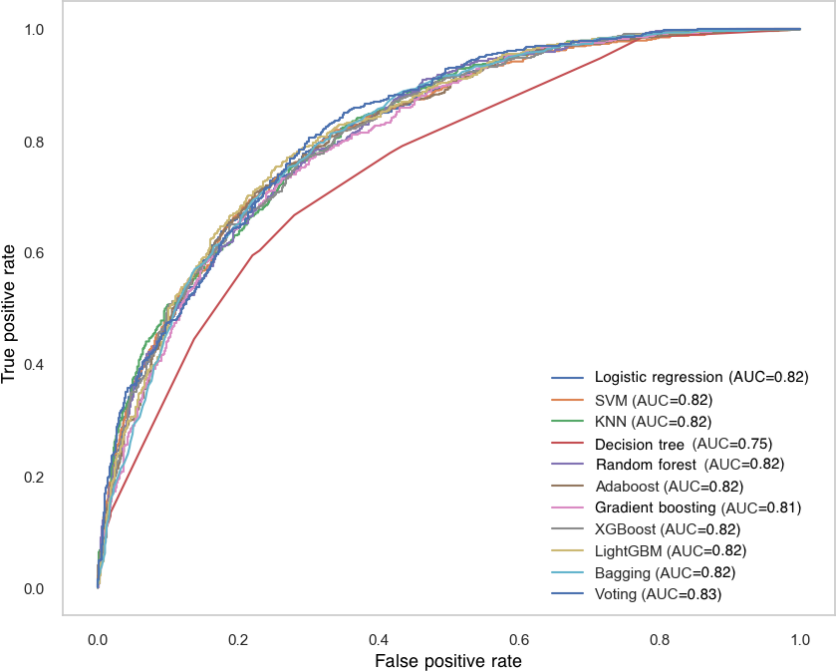
Receiver operating characteristic (ROC) curves and area under the curve (AUC) values for various classification models in predicting mortality outcomes in intensive care unit (ICU) patients with cerebrovascular diseases. KNN: k-nearest neighbors; LightGBM: light gradient boosting machine; SVM: support vector machine.

Eleven ML classifiers were trained and evaluated using the selected features. Table 2 presents the performance metrics, and Figure 2 illustrates ROC curves for each model.

**Table 2.** Performance metrics of machine learning models.

Classifier	Accuracy	ROC AUC^a^	Recall	Precision	*F*_1_-score
Logistic regression	74.18	0.82	0.65	0.73	0.69
SVM^b^	74.31	0.82	0.66	0.73	0.69
KNN^c^	72.29	0.82	0.51	0.78	0.62
Decision tree	69.70	0.75	0.67	0.65	0.66
Random forest	72.92	0.82	0.66	0.70	0.68
AdaBoost	74.12	0.82	0.67	0.72	0.69
Gradient boosting	73.30	0.81	0.65	0.72	0.68
XGBoost^d^	73.42	0.82	0.68	0.70	0.69
LightGBM^e^	74.87	0.82	0.70	0.72	0.71
Bagging	73.93	0.82	0.72	0.70	0.71
Voting	72.98	0.83	0.61	0.73	0.66

a ROC AUC: area under the receiver operating characteristic curve.

b SVM: support vector machine.

c KNN: k-nearest neighbors.

d XGBoost: extreme gradient boosting.

e LightGBM: light gradient boosting machine.

LightGBM demonstrated strong overall performance, achieving an accuracy of 74.87% and an ROC AUC of 0.82, a recall of 0.70, a precision of 0.72, and an *F*_1_-score of 0.71. This model demonstrated robust performance across all evaluation metrics and was highly effective in discriminating between classes and minimizing false positives.

Bagging performed similarly to LightGBM, with an accuracy of 73.93%, ROC AUC of 0.82, recall of 0.72, precision of 0.70, and an *F*_1_-score of 0.71. It also provided balanced performance but with slightly lower precision compared to LightGBM.

XGBoost and AdaBoost showed competitive performance with an accuracy of around 73.5%, both achieving an ROC AUC of 0.82. XGBoost had a recall of 0.68 and *F*_1_-score of 0.69, while AdaBoost exhibited a slightly higher recall of 0.67 but a slightly lower precision (0.72) compared to XGBoost.

SVM and random forest both showed promising performance, with accuracy values of 74.31% and 72.92%, respectively. These models also achieved similar ROC AUC scores of 0.82, with SVM yielding a slightly higher recall of 0.66 compared to random forest (0.66). Both models displayed well-balanced performance, with SVM achieving the highest recall.

Logistic regression, a widely used baseline model for binary classification, achieved an accuracy of 74.18%, an ROC AUC of 0.82, and a recall of 0.65, demonstrating discriminative performance comparable to that of ensemble-based models such as LightGBM and Bagging.

K-nearest neighbors performed well in terms of ROC AUC (0.82) but had a relatively lower recall of 0.51, which affected its *F*_1_-score (0.62). This highlights its ability to discriminate classes well but with a tendency to miss true positive cases.

Decision tree performed the worst, with an accuracy of 69.70%, an ROC AUC of 0.75, and relatively low precision (0.65) and recall (0.67), making it less suitable for this classification task when compared to other models.

After restructuring the experimental pipeline to ensure that imputation and feature selection were performed exclusively on the training data, model performance remained largely stable. The LightGBM model achieved an ROC AUC of 0.81 on the independent test set, indicating that the original performance estimates were not substantially inflated by data leakage.

In summary, ensemble models—particularly LightGBM and Bagging—demonstrated the best balance between sensitivity and specificity.

### Temporal Validation Analysis

To further evaluate the robustness and temporal generalizability of the proposed models, we conducted a temporal validation using anchor_year_group to separate training and testing cohorts. Models trained on earlier admission years and tested on later years demonstrated stable discriminative performance. The LightGBM model achieved an AUC of 0.85, with an accuracy of 0.78, recall of 0.75, and *F*_1_-score of 0.71. Logistic regression showed comparable performance (AUC=0.85), while the Bagging model demonstrated slightly lower discrimination (AUC=0.84). These results suggested that model discrimination remained stable when applied to temporally independent data; however, performance estimates in the temporal test set should be interpreted with caution due to potential under-ascertainment of out-of-hospital mortality in more recent calendar years of the MIMIC-IV database.

### Performance Comparison With Conventional ICU Severity Scores

When evaluated as standalone predictors on the independent test set, conventional ICU severity scores demonstrated moderate discrimination. Simplified Acute Physiology Score II (SAPS-II) achieved the highest performance among traditional scores (ROC AUC=0.73; accuracy=0.65), followed by APS-III (ROC AUC=0.72; accuracy=0.66), Oxford Acute Severity of Illness Score (ROC AUC=0.66), and Logistic Organ Dysfunction System (ROC AUC=0.66). In comparison with these rule-based ICU severity scores, the LightGBM model demonstrated higher discrimination on the same test set (ROC AUC=0.83; accuracy=0.75). DeLong testing confirmed that LightGBM significantly outperformed APS-III as a standalone predictor (ΔAUC=0.12, *P*<.001), indicating meaningful incremental prognostic value beyond conventional ICU severity scoring systems. Notably, when compared with a fully specified multivariable logistic regression model trained on the same feature set, LightGBM showed comparable discriminative performance, highlighting that both linear and nonlinear modeling approaches can achieve similar discrimination in this setting. The added value of ensemble ML models was primarily reflected in calibration performance, DCA, and the ability to capture complex feature interactions. Importantly, this comparable performance persisted under temporal validation, indicating that both linear and nonlinear models maintained stable discrimination when applied to future patient cohorts.

### Confusion Matrix and Calibration Curve

**Figure 3.**
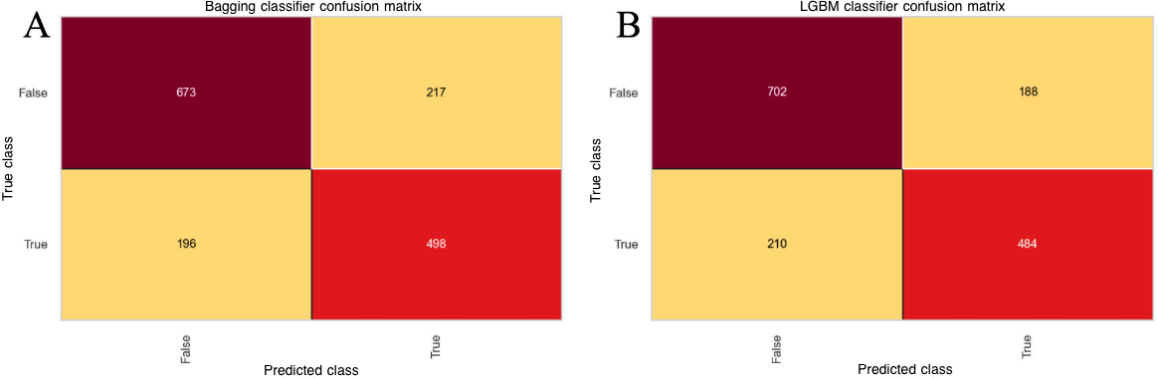
Confusion matrices for the Bagging and light gradient boosting machine (LightGBM) classifier models in predicting mortality outcomes in intensive care unit (ICU) patients with cerebrovascular diseases. (**A**) Confusion matrix for the Bagging classifier model. (**B**) Confusion matrix for the LightGBM classifier model. In the confusion matrices, darker red cells indicate higher counts, whereas lighter yellow cells indicate lower counts.

The confusion matrix for the top-performing models (LightGBM and Bagging) demonstrated high accuracy in predicting both positive (mortality) and negative (survival) outcomes (Figure 3). Bagging exhibited a strong balance between sensitivity and specificity, with a true positive rate of 0.72 and a true negative rate of 0.76.

**Figure 4.**
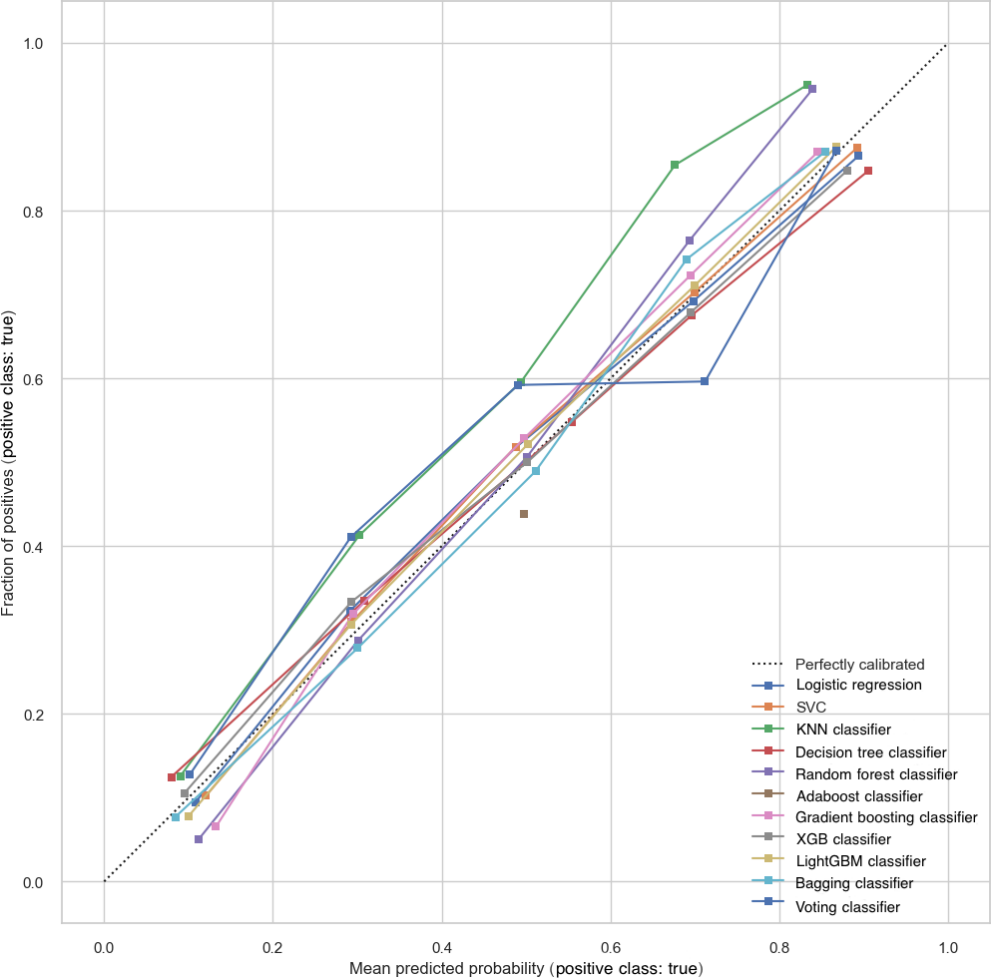
Calibration curves for different classification models in predicting mortality outcomes in intensive care unit (ICU) patients with cerebrovascular diseases. KNN: k-nearest neighbors; LightGBM: light gradient boosting machine; SVC: support vector classifier; XGB: extreme gradient boosting.

Calibration curves (Figure 4) were plotted to evaluate the agreement between predicted and observed mortality risk. Both LightGBM and Bagging demonstrated good calibration, with predicted probabilities closely aligning with actual outcomes across the full risk spectrum. To complement the visual assessment, quantitative calibration metrics were also computed. LightGBM achieved the lowest Brier score (0.171), indicating the best overall probability accuracy, and showed a calibration slope close to 1 (1.14) with a small intercept (0.057), suggesting minimal systematic over- or underestimation. Bagging also demonstrated strong performance with a low Brier score (0.174), a calibration slope of 1.12, and an intercept of 0.021. Together, these results indicate that LightGBM and Bagging provided well-calibrated and reliable mortality risk predictions.

### DCA and Clinical Utility

**Figure 5.**
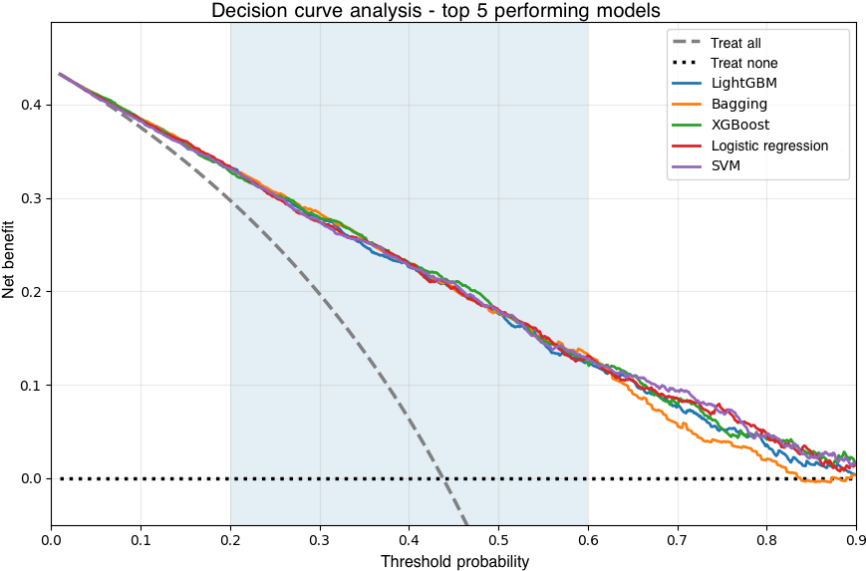
Decision curve analysis (DCA) of the top five machine learning models for mortality prediction. The DCA illustrates the net benefit of using 5 predictive models (light gradient boosting machine [LightGBM], Bagging, extreme gradient boosting [XGBoost], logistic regression, and support vector machine [SVM]) across a range of threshold probabilities. The dashed gray curve represents the “treat all” strategy, which was calculated using the standard DCA definition, whereby the net benefit at a threshold probability of zero equals the observed event prevalence and decreases with increasing threshold probability. The dotted horizontal line represents the “treat none” strategy. Shaded areas indicate clinically relevant threshold probability ranges. A model with a higher net benefit at a given threshold is considered to have greater potential clinical utility.

The clinical utility of the top 5 predictive models was evaluated using DCA. As shown in Figure 5, all models demonstrated a higher net benefit than the “treat none” strategy across a broad range of clinically relevant threshold probabilities. When compared with the “treat all” strategy, the ML models provided additional net benefit primarily at moderate-to-higher threshold probabilities (approximately 0.4‐0.8), whereas at lower thresholds, their net benefit largely overlapped with the “treat all” approach.

Across this clinically relevant range, LightGBM generally showed the most favorable net benefit, with Bagging and XGBoost exhibiting closely comparable performance. Logistic regression and SVM also demonstrated consistent net benefit above the baseline strategies. Overall, these findings indicate that the proposed models may be most useful for guiding more selective clinical decisions—such as identifying high-risk patients for intensified monitoring or intervention—rather than for low-threshold scenarios where treating all patients may already be appropriate.

### SHAP Analysis and Feature Importance

We performed SHAP analysis to interpret individual features’ influence on the model’s predictions. SHAP values provide a quantitative way to understand how much each feature contributes to the model’s predictions. We used 2 complementary visualizations—a SHAP bar plot and a layered violin plot—to explore global feature importance and the detailed impact of each feature.

### Key Insights From SHAP Analysis

**Figure 6.**
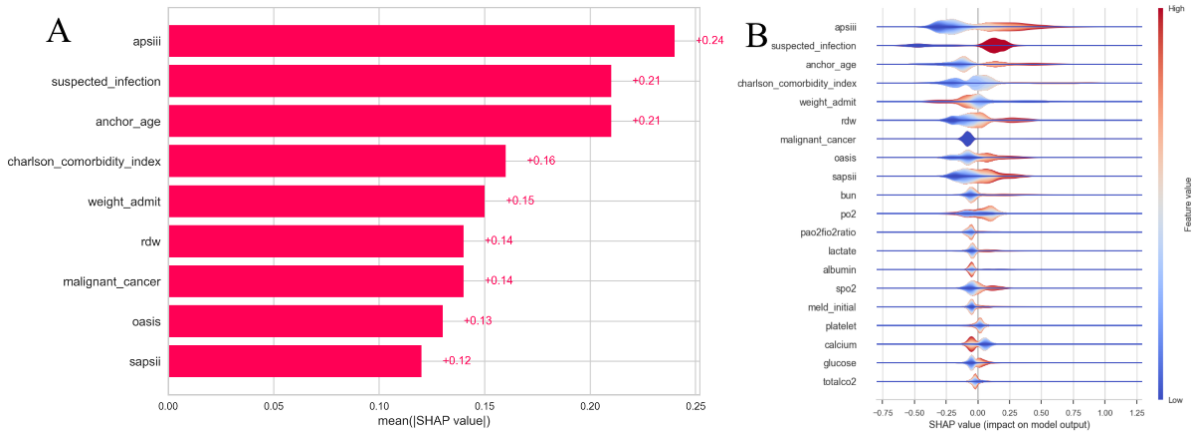
Shapley additive explanation (SHAP) plots illustrating the impact of key features on model predictions. (**A**) Bar plot showing the average absolute SHAP values for the most influential features. (**B**) Layered violin plot displaying the distribution of SHAP values for the top features, with color indicating feature values (red for high, blue for low).

The SHAP bar plot (Figure 6A) and layered violin plot (Figure 6B) both highlight the most influential features in the model. The bar plot shows the average absolute SHAP values for the top features, while the violin plot reveals the distribution of SHAP values for each feature, providing a more nuanced view of how each feature impacts the model’s predictions. For clarity of visualization, the aggregated “sum of remaining features” term was excluded from the SHAP bar plot, allowing direct comparison of the relative importance of individual predictors.

The top 5 features with the highest average SHAP values are as follows (key observations):

- “apsiii” (acute physiology score III) is the most important feature, with an average SHAP value of 0.24 (SD 0.17). This feature has a broad range of SHAP values, both positive and negative, indicating its complex impact on the model’s output depending on the severity of the patient’s condition.
- “suspected_infection” and “anchor_age” both have a mean SHAP value of 0.21 (SD 0.19 and 0.15, respectively), signifying their strong positive influence on the model’s predictions. Higher values of suspected_infection (indicating suspicion of infection) and anchor_age (older age) are associated with a higher likelihood of adverse outcomes.
- “charlson_comorbidity_index” shows a more balanced SHAP value distribution (mean SHAP value of 0.16, SD 0.19), suggesting that comorbidities have a mixed influence on the model, with both positive and negative impacts depending on the values of the index.
- “weight_admit” and “rdw” both show a strong positive contribution to the model, with average SHAP values of 0.15 (SD 0.16) and 0.14 (SD 0.13), respectively. Higher values for these features correlate with a higher probability of adverse outcomes.
- “malignant_cancer” and “oasis” also demonstrate strong positive contributions, with SHAP values predominantly concentrated in the positive range.

### Feature Impact Analysis

The layered violin plot (Figure 6B) provides a deeper understanding of how the distribution of feature values influences the model’s predictions. The plot shows that the SHAP values for most of the top features are concentrated in the positive range, indicating that higher values for these features generally lead to higher predicted risks. For example, “suspected_infection,” “anchor_age,” and “weight_admit” all have SHAP values that are predominantly positive, meaning that higher values of these features are associated with a higher likelihood of adverse outcomes.

However, some features, such as “apsiii” and “charlson_comorbidity_index,” show more variability in their SHAP values, with both positive and negative values present. This indicates that these features have a more complex relationship with the model’s predictions, contributing in both directions depending on the specific values.

Overall, the visualizations help us understand which features are most important in the model’s decision-making process and how the value of each feature affects the predictions.

### Summary of Key Findings

This study analyzed 5280 ICU patients with cerebrovascular diseases and found that older age, higher comorbidity scores, suspected infection, and dementia were associated with higher mortality. Feature selection identified 35 significant factors, and ML models were trained using these features. LightGBM and Bagging performed best, with LightGBM achieving 74.87% accuracy and an ROC AUC of 0.82. SHAP analysis highlighted key features such as acute physiology score III, suspected infection, and age as the most influential predictors of mortality. These models offer reliable tools for predicting all-cause mortality as recorded in the MIMIC-IV database and identifying high-risk patients in critical care settings.

## Discussion

### Principal Findings

This study demonstrates the potential of ML models to predict all-cause mortality among ICU patients with cerebrovascular diseases, using mortality outcomes as recorded in the MIMIC-IV database. Recent studies have shown that ML approaches can enhance prognostic accuracy in stroke and cerebrovascular disease populations, outperforming traditional clinical scores and improving individualized risk stratification [11,12]. Using the extensive MIMIC-IV database, we developed and evaluated several ML algorithms, identifying LightGBM and Bagging models as top performers across multiple metrics, consistent with the findings from recent ML-based stroke outcome prediction research [13]. These findings highlight the robustness of these models and underscore the value of ML in supporting clinical decision-making for critically ill patients with complex cerebrovascular conditions [14].

### Key Findings and Clinical Implications

Critical predictors of all-cause mortality identified in this cohort included suspected infection, age, Charlson comorbidity index, and malignancy. These factors align with prior research emphasizing the influence of comorbidities and infections on stroke outcomes [15,16]. Notably, suspected infection was highly prevalent in this cohort (73.4%), reflecting the high burden of infectious complications among critically ill patients with cerebrovascular disease admitted to the ICU, emphasizing the need for the timely recognition and management of infections—especially pneumonia—which significantly worsen stroke prognosis [17,18]. Age remains a well-established prognostic factor, with older patients facing higher mortality risk [19]. The Charlson comorbidity index further confirms the elevated risk among patients with multiple comorbid conditions, such as diabetes and hypertension [20]. SHAP analysis elucidated the relative importance of these variables, providing actionable insights for clinicians during risk assessment.

The SHAP analysis highlighted APS-III, suspected infection, comorbidity burden, and age as the most influential predictors of mortality, which is consistent with established stroke prognostic literature [21]. APS-III reflects the overall physiological severity on ICU admission, and higher scores have been strongly associated with increased mortality risk in critically ill stroke patients [22]. Infection status—particularly early respiratory or systemic infections—is a well-recognized complication that substantially increases mortality risk following ischemic stroke [23]. Similarly, a higher Charlson comorbidity index captures chronic disease burden, such as cardiovascular disease, diabetes, or malignancy, all of which worsen poststroke prognosis [24]. Age is one of the most robust and widely validated predictors of mortality and functional decline after ischemic stroke. The prominence of these variables in our SHAP results reinforces the biological plausibility of the model and indicates apparent alignment with clinical knowledge, supporting the reliability and interpretability of the ML predictions [25].

It is noteworthy that “suspected infection” was defined based on clinical interventions, including antibiotic administration and body fluid culture collection, rather than solely on physiological confirmation. As such, this feature may partially reflect clinicians’ perception of illness severity and concern for systemic infection, functioning as a process-of-care proxy rather than an independent biological marker. The considerable contribution of this variable in SHAP analyses suggests that the model may be learning from real-world clinical decision-making patterns embedded in ICU workflows. This characteristic is inherent to retrospective critical care datasets and highlights the importance of cautious interpretation when incorporating intervention-derived features into predictive models.

It should be noted that APS-III is a composite severity score derived from age and multiple physiological variables [26]. In the present models, APS-III was included alongside individual components, such as anchor_age and specific physiological measurements (eg, blood urea nitrogen and heart rate). Consequently, collinearity exists between APS-III and its constituent variables, and the contribution of age and physiological severity may be distributed across both the composite score and individual features in the SHAP-based importance rankings. Therefore, the prominence of APS-III in the SHAP analysis should be interpreted as reflecting overall illness severity rather than an independent effect separate from demographic or physiological factors, and the relative importance of these variables should be considered collectively.

Although established ICU severity scores such as APS-III and SAPS-II demonstrated reasonable predictive performance, the proposed ML models demonstrated higher discrimination than individual conventional ICU severity scores, while showing comparable performance to multivariable logistic regression models trained on the same feature set. Notably, APS-III, which represents one of the most comprehensive and widely used severity scores in critical care, was identified as the most influential individual predictor in the SHAP analysis.

Despite this, the LightGBM model provided a statistically significant improvement over APS-III alone, suggesting that the ML approach integrates complementary clinical information beyond conventional scoring systems. These findings support the role of ML as an enhancement to, rather than a replacement for, traditional ICU risk stratification tools.

Our results suggest that ML models, particularly LightGBM and Bagging, offer fairly accurate, individualized mortality predictions, enabling ICU clinicians to identify high-risk patients early. This capability could facilitate better resource allocation and more targeted interventions, ultimately improving patient outcomes where timely decision-making is critical.

In addition to predictive accuracy, the practical integration of the proposed models into clinical workflows is an important consideration. Because all input features used by the LightGBM and Bagging models are routinely collected in the ICU and are available within electronic health records, the models could be embedded into existing clinical decision support systems to generate real-time mortality risk estimates. Such integration would allow clinicians to identify high-risk patients shortly after ICU admission, prioritize monitoring intensity, optimize resource allocation, and guide discussions regarding prognosis. The use of SHAP-based explanations further enhances transparency by enabling clinicians to understand the key factors driving each patient’s prediction. Future work will focus on implementing and evaluating these models in prospective clinical settings to assess usability, workflow impact, and real-world clinical benefit.

### Comparison With Existing Literature

Consistent with previous studies, our findings indicate that ML models can provide incremental prognostic value beyond conventional bedside severity scores for ICU patients with stroke-related conditions [27]. In our cohort, commonly used ICU severity scores showed moderate discrimination when evaluated as standalone predictors on the same independent test set (eg, SAPS-II AUC=0.73; APS-III AUC=0.72; Oxford Acute Severity of Illness Score AUC=0.66; Logistic Organ Dysfunction System AUC=0.66). In contrast, the best-performing ML model (LightGBM) achieved substantially higher discrimination (AUC=0.83), and DeLong testing confirmed a statistically significant improvement over APS-III alone (ΔAUC=0.12, *P*<.001). These results suggest that the ML approach integrates complementary information beyond established scoring systems, supporting enhanced early risk stratification.

Our focus on patients with intracranial artery stenosis or occlusion extends the literature by providing detailed insights into this understudied subgroup. The large, publicly available MIMIC-IV dataset enabled robust analysis of more than 5000 patients, improving generalizability. The inclusion of mortality occurring after hospital discharge, as captured in the MIMIC-IV database, and a broad set of early clinical features, including biochemical markers and physiological severity indices, provide a comprehensive assessment of mortality risk compared with many prior studies limited by smaller samples or narrower feature sets.

Beyond traditional performance metrics, DCA demonstrated the clinical utility of the proposed models under specific decision thresholds. As shown in Figure 5, LightGBM and Bagging provided higher net benefit than the “treat none” strategy across a broad range of threshold probabilities and demonstrated clear advantage over the “treat all” strategy primarily at moderate-to-high risk thresholds. This pattern is expected given the relatively high prevalence of the outcome and indicates that the models may be particularly useful for guiding more selective clinical decisions.

In this context, incorporating these models into ICU workflows could support improved resource allocation by more accurately identifying patients at higher risk who may benefit from intensified monitoring or intervention. Together with model interpretability using SHAP, these findings support the potential for targeted, real-world application of our ML approach rather than indiscriminate use across all clinical scenarios.

Importantly, the robustness of the findings was further supported by temporal validation. When models were trained on earlier admission years and evaluated on later years, performance remained stable, with no meaningful degradation in discrimination or calibration. This temporal evaluation reduces concerns regarding information leakage inherent to random splitting in long-term retrospective datasets and supports the potential real-world applicability of the proposed models in contemporary ICU populations.

### Strengths and Limitations

Key strengths include the use of multiple advanced feature selection methods (gradient boosting classifier, BorutaShap, RFECV) to isolate the most relevant predictors and the integration of SHAP for model interpretability, which fosters clinical trust and facilitates implementation.

Mortality status in this study was determined solely based on the presence or absence of a recorded dod in the MIMIC-IV database. As a result, deaths occurring outside the capture scope of the database—particularly out-of-hospital deaths in more recent calendar years—may not have been recorded. Accordingly, the absence of a documented dod should not be interpreted as confirmed long-term survival. Because mortality ascertainment relied on registry-based death records rather than active longitudinal follow-up, longer-term outcomes, such as functional recovery or late mortality beyond what was captured in the database, could not be assessed.

A related observation in this cohort was the marked discrepancy between in-hospital mortality and overall recorded mortality, indicating that a substantial proportion of deaths occurred after hospital discharge. In critically ill patients with advanced age, high comorbidity burden, malignancy, or severe physiological derangement, such postdischarge mortality may reflect transitions to hospice or comfort-focused care rather than unexpected physiological deterioration alone. Because explicit indicators of goals-of-care decisions—such as do not resuscitate status or comfort measures only orders—are not consistently captured in the public MIMIC-IV database, these factors could not be directly modeled. Consequently, the predictive models may partially capture clinical trajectories associated with end-of-life decision-making in addition to underlying illness severity. This limitation is particularly relevant in the context of temporal validation, as the incomplete capture of out-of-hospital mortality in more recent calendar years may introduce outcome misclassification, potentially inflating apparent model performance when the absence of a recorded death reflects missing data rather than true survival.

Moreover, all predictors were derived exclusively from the data available within the first 24 hours of ICU admission, prior to most formal goals-of-care decisions. However, the MIMIC-IV database does not provide reliable “present on admission” indicators for stroke diagnoses. As a result, some patients may have developed ischemic stroke after ICU admission rather than being admitted primarily for acute stroke, introducing potential temporal misclassification between disease onset and feature extraction. This limitation may affect the specificity of the cohort and should be considered when interpreting the model’s applicability to strictly acute stroke populations. This design suggests that the models primarily reflect early physiological severity and comorbidity burden rather than downstream treatment choices. From a clinical perspective, the early identification of patients at extremely high risk of mortality—regardless of whether death occurs following aggressive treatment or comfort-focused care—remains highly relevant for prognostic communication, care planning, and resource allocation in the ICU.

Another important limitation is the absence of direct measures of neurological stroke severity, such as the National Institutes of Health Stroke Scale (NIHSS), which is not systematically available in the MIMIC-IV database. Without the NIHSS, the model cannot distinguish between patients with mild neurological deficits and severe systemic illness versus those with large, devastating strokes. Accordingly, the model may be better interpreted as predicting mortality among critically ill patients with stroke-associated conditions (“death with stroke”) rather than mortality driven exclusively by cerebrovascular injury itself (“death from stroke”). The prominence of APS-III and other systemic physiological markers in the SHAP analysis supports this interpretation and reflects the clinical reality of ICU stroke populations, in whom outcomes are often driven by multiorgan dysfunction and comorbidity burden. Therefore, the proposed model should not be viewed as a replacement for stroke-specific prognostic tools used in acute neurological decision-making, but rather as a complementary instrument for global mortality risk stratification in critically ill patients with stroke-associated conditions.

In addition, the feature “suspected infection” was operationally defined based on clinical actions (antibiotic administration and body fluid culture collection) rather than microbiological confirmation alone. As such, this variable may partially act as a proxy for clinician concern or illness severity, introducing potential circularity whereby the model learns from care processes rather than purely independent physiological risk factors. This reflects real-world ICU practice but warrants cautious interpretation of its predictive contribution. Although race and ethnicity were available in the database and are reported in the descriptive characteristics, this study did not perform formal subgroup or fairness analyses to evaluate potential differential model performance across demographic groups. Given known disparities in critical care delivery and the use of process-of-care–derived features such as suspected infection, algorithmic bias cannot be excluded and should be addressed in future validation studies.

The final key limitation of this study is the absence of an external validation cohort. Although rigorous internal validation was performed using an independent hold-out test set, the generalizability of the model to other institutions, populations, and clinical environments remains uncertain. Moreover, all data were derived from a single academic medical center, and institutional practices regarding discharge disposition, hospice referral, and comfort-focused care may differ substantially across health systems. As a result, the model may partially reflect center-specific end-of-life care workflows, potentially limiting its applicability to settings with different palliative care practices. At the time of this study, no external ICU dataset contained sufficiently detailed information on intracranial artery stenosis or occlusion together with reliable mortality outcomes to enable external validation. Future studies should validate these findings in multicenter datasets or prospective clinical cohorts.

### Future Directions

Future research should aim to integrate these ML models into clinical workflows for real-time risk stratification. The incorporation of additional data types, such as imaging and genetic information, may further improve predictive accuracy. Prospective validation in diverse clinical settings is needed to confirm generalizability and assess impact on patient outcomes. Combining ML models with established clinical scores like NIHSS and sequential organ failure assessment could enhance predictive power and support decision-making.

In conclusion, this study supports the use of ML models to provide personalized mortality risk assessments for ICU patients with cerebrovascular disease associated with intracranial artery stenosis or occlusion, with potential to improve critical care management and patient prognosis.

### Conclusions

ML models, especially LightGBM and Bagging, demonstrate comparable performance in predicting all-cause mortality in ICU patients with cerebrovascular diseases involving intracranial artery stenosis or occlusion. By leveraging routinely collected clinical data and identifying key risk factors, such as suspected infection, age, and comorbidities, these models offer valuable support for early risk stratification and clinical decision-making in critical care. Further external validation and real-world implementation studies are warranted to confirm their generalizability and clinical impact.

## Supplementary material

10.2196/82042 Multimedia Appendix 1 Pearson correlation matrix of the final selected features.

## Abbreviations

AdaBoost: adaptive boosting
DCA: decision curve analysis
dod: date of death
*ICD-9/10*: *International Classification of Diseases, Ninth and Tenth Revision*
ICU: intensive care unit
LightGBM: light gradient boosting machine
MIMIC-IV: Medical Information Mart for Intensive Care IV
ML: machine learning
NIHSS: National Institutes of Health Stroke Scale
RFECV: recursive feature elimination with cross-validation
ROC AUC: area under the receiver operating characteristic curve
SAPS II: Simplified Acute Physiology Score II
SHAP: Shapley additive explanation
SVM: support vector machine
XGBoost: extreme gradient boosting
